# Pretransplant Immune- and Apoptosis-Related Gene Expression Is Associated with Kidney Allograft Function

**DOI:** 10.1155/2016/8970291

**Published:** 2016-06-12

**Authors:** Dorota Kamińska, Katarzyna Kościelska-Kasprzak, Paweł Chudoba, Oktawia Mazanowska, Mirosław Banasik, Marcelina Żabinska, Maria Boratyńska, Agnieszka Lepiesza, Agnieszka Gomółkiewicz, Piotr Dzięgiel, Marian Klinger

**Affiliations:** ^1^Department of Nephrology and Transplantation Medicine, Wroclaw Medical University, 52-129 Wrocław, Poland; ^2^Department of Vascular, General and Transplant Surgery, Wroclaw Medical University, 52-129 Wrocław, Poland; ^3^Department of Histology and Embryology, Wroclaw Medical University, 50-368 Wrocław, Poland

## Abstract

Renal transplant candidates present immune dysregulation, caused by chronic uremia. The aim of the study was to investigate whether pretransplant peripheral blood gene expression of immune factors affects clinical outcome of renal allograft recipients.* Methods*. In a prospective study, we analyzed pretransplant peripheral blood gene expression in87 renal transplant candidates with real-time PCR on custom-designed low density arrays (TaqMan).* Results*. Immediate posttransplant graft function (14-day GFR) was influenced negatively by* TGFB1* (*P* = 0.039) and positively by* IL-2* gene expression (*P* = 0.040). Pretransplant blood mRNA expression of apoptosis-related genes (*CASP3*,* FAS, *and* IL-18*) and Th1-derived cytokine gene* IFNG* correlated positively with short- (6-month GFR* CASP3*: *P* = 0.027,* FAS*: *P* = 0.021, and* IFNG*: *P* = 0.029) and long-term graft function (24-month GFR* CASP3*: *P* = 0.003,* FAS*: *P* = 0.033,* IL-18*: *P* = 0.044, and* IFNG: P* = 0.04).* Conclusion*. Lowered pretransplant Th1-derived cytokine and apoptosis-related gene expressions were a hallmark of subsequent worse kidney function but not of acute rejection rate. The pretransplant* IFNG* and* CASP3* and* FAS* and* IL-18* genes' expression in the recipients' peripheral blood is the possible candidate for novel biomarker of short- and long-term allograft function.

## 1. Introduction

Kidney transplantation outcome depends on many immunological and nonimmunological factors. Renal transplant candidates present immune dysregulation, caused by chronic uremia followed by renal replacement therapy [1]. Recently, uremia was reported to influence cytokine synthesis [2]. While the immune response shift is well described in the literature, still it is not clear whether the pretransplant chronic inflammation affects posttransplant outcome. From routine blood tests CRP (C-reactive protein) and albumin levels were shown not only to reflect the general immune response in uremic patients [3] but also to predict kidney outcome [4, 5].

Pretransplant cytokine profiles were also considered to exert a deleterious effect on graft function or survival, but the published data have been inconclusive [6, 7].

The aim of the study was to investigate whether pretransplant peripheral blood gene expression affects clinical outcome of renal allograft recipients. In a prospective study we analyzed a wide range of immune factors known to be related to inflammation (IL-6, IL-8, NGAL, and TNF-alpha), apoptosis (Fas, caspase 3, p53, and IL-18), and lymphocyte T activation (IFN-gamma, IL-2) as well as regulatory T cell (IL-10, TGF-beta, and Foxp3) function with the real-time PCR method. Pretransplant cytokine gene expression linked to recipient- and donor-related factors was examined with further analysis of allograft function. The working hypothesis was the search for the novel predictive biomarkers connecting the recipients' pretransplant immune and inflammatory status with transplant outcome.

## 2. Patients

The study was carried out prospectively on 87 low risk consecutive renal transplant recipients (aged 16–72 years, mean 47 years; 34 females, 53 males) transplanted between 2006 and 2012 at Wroclaw Medical University. Among them 66 patients were treated with hemodialysis and 21 patients with peritoneal dialysis. They remained on renal replacement therapy from 1 to 97 months (mean 25 ± 18 months).

The patients received organs from donors (81 deceased, 6 living) aged from 16 to 72 years. Eighty-three recipients received their first graft, while for 4 of them it was the second transplant. They were being transplanted after cold ischemia time from 9 to 45 hours. Three recipients presented pretransplant panel reactive antibodies (PRA) above 20%.

Immunosuppressive therapy consisted of corticosteroids with cyclosporine and mycophenolate mofetil/sodium in 35 cases, corticosteroids with tacrolimus and mycophenolate mofetil/sodium in 49 cases, corticosteroids with everolimus and tacrolimus in 1 case, and corticosteroids with sirolimus and cyclosporine in 2 cases. Four recipients received induction therapy with anti-CD25 antibodies at the time of transplantation. Donor and recipient characteristics are presented in Table 1.

Pretransplant demographic and clinical data (age, gender, weight, height, comorbidity, and history of chronic kidney disease) were obtained from medical records from patients' dialysis centers. Posttransplant clinical data (renal function, immunosuppressive therapy, complications, and outcome) were collected from medical records of the transplant outpatient clinic of the Department of Nephrology and Transplantation Medicine in Wroclaw, Poland. Estimated glomerular filtration rate (eGFR) was calculated using the abbreviated Modification of Diet in Renal Disease study formula (MDRD).

Blood samples for routine laboratory tests assessing pretransplant inflammatory response as well as samples for gene expression were collected immediately before the transplant procedure and introduction of immunosuppressive therapy. Posttransplant laboratory tests were completed during routine visits to an outpatient clinic.

The study was approved by the Commission of Bioethics at Wroclaw Medical University, and all aspects of the study were in accordance with the World Medical Association Declaration of Helsinki. Before enrollment, each patient read an information sheet and provided fully informed consent.

## 3. Methods

The peripheral blood samples were obtained with PAXgene Blood RNA tubes. RNA was isolated with a PAXgene Blood RNA kit (PreAnalytics) and reversely transcribed with High Capacity RNA to cDNA kit (Applied Biosystems). The peripheral blood gene expression of caspase-3 (*CASP3*, Hs00263337_m1), Fas (*FAS*, Hs00236330_m1), p53 (*TP53*, Hs00153349_m1), Foxp3 (*FOXP3*, Hs00203958_m1), IFN-gamma (*IFNG*, Hs00174143_m1), interleukin-2 (*IL-2*, Hs00174114_m1), interleukin-6 (*IL-6*, Hs00174131_m1), interleukin-8 (*IL-8*, Hs00174103_m1), interleukin-10 (*IL-10*, Hs00174086_m1), interleukin-17 (*IL-17A*, Hs99999082_m1), interleukin-18 (*IL-18*, Hs00155517_m1), NGAL (*LCN2*, Hs00194353_m1), TGF-beta (*TGFB1*, Hs99999918_m1), and TNF-alpha (*TNF*, Hs00174128_m1) was assessed with real-time PCR on custom-designed low density arrays (TaqMan) with TaqMan PCR Master Mix. All procedures were performed according to the manufacturer's protocols.

GAPDH was chosen as the reference gene, and it was proved not to be related to transplant outcome (delayed graft function (DGF)/acute rejection occurrence (AR), serum creatinine, and GFR).

Each gene was tested in triplicate; raw expression data were averaged and converted to ΔCt, where ΔCt = Ct_gene_ − Ct_GAPDH_ and Ct is the cycle threshold value and defines the calculated cycle number, in which the fluorescence measured during PCR reaction increases over the preset threshold value.

The expression data were analyzed separately for each of the genes in the subsequent manner. The samples with the highest quartile of ΔCt values (which corresponds to the lowest expression level) were chosen as a reference group (ref) for data normalization. The expression data throughout the paper are presented as ΔΔCt = mean ΔCt_ref_ − ΔCt_sample_.

### 3.1. Statistical Analysis

Statistical analysis was performed with the Statistica v.12 statistical package (StatSoft, Poland). The numerical variables were tested for outliers and normality and presented as mean ± standard deviation or median + interquartile range. In case of normally distributed variables, the Student *t*-test and regression were applied. If the normality assumption was not fulfilled, Mann-Whitney *U*-test and Spearman correlation were used. For normally distributed continuous variables, their influence on the outcome was corrected by multiple regression for the other parameters shown significantly in univariate analysis. *P* < 0.05 was considered significant.

## 4. Results

### 4.1. Posttransplant Outcome

After transplantation, immediate kidney function was observed in 77% of the recipients. Delayed graft function (defined as a need of hemodialysis in the first week after transplantation) was seen in 20 cases (23%). Seventeen recipients (19%) experienced at least one biopsy-proven rejection episode within the first posttransplant year. Recipients were followed up for 5 years after transplantation. During that time 5 recipients died (5.7%) and 11 lost their grafts (12%). Severe cardiovascular complications occurred in 7 recipients (8%), serious infections with a need of hospitalization occurred in 10 cases (11%), 13 (15%) recipients suffered from CMV disease, and 18 recipients (21%) presented recurrent urinary tract infections. There was one case of malignant tumor.

After 24 months of observation 36% of recipients presented excellent kidney function with GFR above 50 mL/min (mean GFR 60.3 ± 5.4 mL/min).

### 4.2. Pretransplant Clinical Parameters and Kidney Allograft Outcome

Recipients and donors characteristics are included in Table 1 and the clinical pretransplant recipient blood parameters are presented in Table 2. All the parameters were analyzed in relation to graft short- and long-term outcome.

The pretransplant blood parameters (CRP, albumin, cholesterol, complete blood cell test, etc.) did not affect posttransplant outcome or acute rejection episodes. Time and type of previous renal replacement therapy—hemodialysis or peritoneal dialysis—did not influence outcome of transplantation. Moreover, in our study group, we found no association of cold ischemia time or number of HLA mismatches and graft function or survival.

From the clinical parameters studied only donor age, recipient age, and recipient BMI proved to influence allograft outcome.

Both recipients' and donors' age were negatively correlated with immediate and short- and long-term allograft function at any time point studied. Donor age was also a risk factor for delayed graft function and acute rejection. All significant correlations of clinical parameters with graft function are summarized in Table 4.

Recipients' age was associated with worse graft function from 14 days to 24 months (14-day GFR *r* = −0.33, *P* = 0.003; 1-month GFR *r* = −0.37, *P* = 0.001; 3-month GFR *r* = −0.31, *P* = 0.0; 6-month GFR *r* = −0.39, *P* = 0.001; 12-month GFR *r* = −0.33, *P* = 0.008; and 24-month GFR *r* = −0.35, *P* = 0.012).

Donor age was a strong risk factor for graft function deterioration from the beginning (14-day GFR *r* = −0.33, *P* = 0.004; 1-month GFR *r* = −0.41, *P* < 0.001; and 3-month GFR *r* = −0.44, *P* < 0.001) up to 24 months (6-month GFR *r* = −0.60, *P* < 0.001; 12-month GFR *r* = −0.48, *P* < 0.001; and 24-month GFR *r* = −0.55, *P* < 0.001). Donor age was also a risk factor for delayed graft function (52.2 ± 10.4 versus 42.8 ± 13.8; *P* = 0.001) and acute rejection (52.1 ± 15.0 versus 43.3 ± 13.0; *P* = 0.018).

Recipients' pretransplant BMI was negatively correlated with the short-term graft function from 3 up to 12 months (3-month GFR *r* = −0.35, *P* = 0.009; 6-month GFR *r* = −0.36, *P* = 0.010; and 12-month GFR *r*_*s*_ = −0.30, *P* = 0.033).

**Figure 1:**
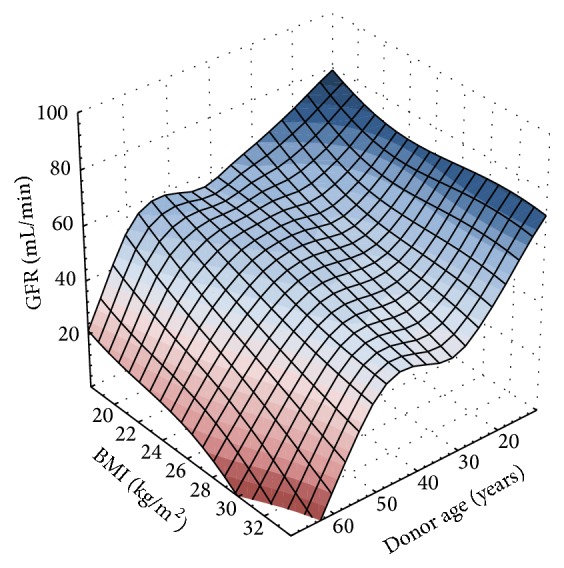
Best-fit surface representation of donor age and recipient BMI influence on graft function 6 months after transplantation.

The correlation between the strongest clinical factors as donor age and recipient BMI with short-term kidney function (expressed by 6-month GFR) is presented in Figure 1.

### 4.3. Gene Expression

Gene expression in the peripheral blood stabilized in PaxBlood RNA tubes was studied with low density TaqMan arrays in triplicate. The observed expression levels are presented in Table 3.* IL-17* gene expression was undetectable in all patients. Besides* IL-2* all other genes expression values were normally distributed. The impact of* IL-2* gene expression on graft function was analyzed with nonparametric statistics.

### 4.4. Correlations between Clinical Pretransplant Parameters and Gene Expression

No influence of recipient gender on gene expression was observed. Pretransplant white blood cell (WBC) affected expression of* FOXP3*,* IL-2*,* LCN2*, and* TNF*. Albumin level affected* IL-10* expression. BMI was strongly correlated with* LCN2* expression.

Significant correlations between clinical pretransplant parameters and gene expression are presented in Table 4.

### 4.5. Gene Expression Association with Graft Function: Univariate Analysis

We observed the association of some gene expression with subsequent graft function (Table 5).

### 4.6. Immediate Posttransplant Graft Function

*TGFB1* exerted a negative influence on short-term graft function (14-day GFR *r* = −0.24, *P* = 0.039). Pretransplant* IL-2* gene expression positively correlated with kidney function in the immediate posttransplant period (14-day GFR *r*_*s*_ = 0.24, *P* = 0.040).

### 4.7. Short-Term Graft Function

*LCN2* exerted a negative influence on short-term graft function (3-month GFR *r* = −0.27, *P* = 0.024). From Th1-derived cytokines,* IFNG* was significantly upregulated in recipients with good kidney function at 6 months after KTx (*r* = 0.27, *P* = 0.029). From apoptosis-related genes* CASP3* and* FAS* were positively correlated with short-term graft function (*CASP3*: 6-month GFR *r* = 0.27, *P* = 0.027;* FAS*: 6-month GFR *r* = 0.28, *P* = 0.021).

### 4.8. Long-Term Graft Function

The long-term kidney function described by eGFR from 12 months to 24 months was positively correlated with pretransplant expression of proapoptotic genes* CASP3* and* FAS* (*CASP3*: 12-month GFR *r* = 0.3, *P* = 0.016, 24-month GFR *r* = 0.41, *P* = 0.003, and* FAS*: 24-month GFR *r* = 0.3, *P* = 0.033). 60 months after transplantation* CASP3* and* IFNG* association was still observed (*CASP3* 60-month GFR: *r*_*s*_ = 0.37, *P* = 0.037;* IFNG* 60-month GFR: *r*_*s*_ = 0.27, *P* = 0.024). Also* IL-18* expression correlated positively with 5-year graft function (60-month GFR *r*_*s*_ = 0.39, *P* = 0.024). The fourth examined proapoptotic gene,* TP53*, did not correlate with graft function at any time point.

From Th1-derived cytokines,* IFNG* was significantly downregulated in recipients with bad clinical outcome from 12 to 24 months after KTx (12-month GFR: *r* = 0.3, *P* = 0.018, 24-month GFR *r* = 0.29, *P* = 0.04). Also pretransplant* IL-2* gene expression positively correlated with kidney function in the late period (18-month GFR *r*_*s*_ = 0.28, *P* = 0.049; 60-month GFR *r*_*s*_ = 0.41, *P* = 0.026, resp.).

We noted no association of expression of other examined genes with graft function at any time point. No correlation was observed between gene profiles and delayed graft function or acute rejection episode rates.

### 4.9. Gene Expression and Pretransplant Clinical Factors Association with Graft Function: Multivariate Analysis

As the particular time point GFR was influenced by a number of continuous variables, both clinical parameters (donor age, recipient age, and recipient BMI) and gene expression (*CASP3*,* FAS*,* IFNG*,* IL-18*,* TGFB1*, and* LCN2*) multiple regression tests were performed. For each time point the parameters presenting significant correlation in simple regression analysis were included in multiple regression model as summarized in Table 5.

From the clinical factors donor age was the strongest risk factor for graft function deterioration from the beginning up to 24 months. Also recipient age was proved to be significant up to 12 months, and an association with BMI was shown 6 months after KTx.

*TGFB1* gene expression was significantly and independently of donor and recipient age associated with the short-term allograft function (14 days).* IFNG* association with 6-month GFR was shown independently from donor age as well as recipient age and BMI. This association was still observed 12 months after KTx. The proapoptotic* CASP3* was shown to be associated positively with long-term graft function (12 and 24 months after KTx) independently from donor age. The association of other apoptosis-accelerating genes—*FAS* and* IL-18*—was seen 24 months after KTx. The impact of* LCN2* seen after 3 months in univariate analysis was not confirmed in multiple regression.

We also studied whether any of the expression levels of the three candidate genes:* CASP3*,* FAS*, and* IL-18* could predict better long-term allograft function (eGFR >50 mL/min 24 months after KTx). The results of ROC analysis are shown in Table 6. All of the three genes presented significant predictive value. Each of* CASP3*,* FAS*, and* IL-18* gene expression levels above cut-off proved to have around 70% sensitivity and at least 80% specificity for good long-term outcome prediction.

## 5. Discussion

We studied the immune background of renal transplant candidates just before the transplantation and its impact on the transplant outcome. We analyzed the expression of immune-related genes at the mRNA level with real-time PCR methodology. We observed that Th1 derived cytokines (IL-2 and IFN-gamma) were significantly correlated with better short- and long-term allograft function. We also observed a positive correlation between pretransplant expression of three proapoptotic genes,* CASP3*,* FAS*, and* IL-18*, with allograft function. Both of the observations were not published before. In addition, it is possible to hypothesize that some level of Th1 constitutive activation supports more active apoptosis which in turn is associated with secretion of anti-inflammatory, tolerogenic molecules as IL-10 and TGF-beta. Collectively, these observations could be interpretative as a credit for hypothesis that some basal immune system cell activation is needed to make them more sensitive to effective inhibition by immunosuppressive drugs.

Chronic kidney disease with progressive uremia is associated with both inflammation and immune deficiency. Primary activation of the innate immune system was noted. It was caused by abnormal function of regulatory T-cells, the depletion of dendritic cells, naive and central memory T cells and B cells, and impaired phagocytic function of polymorphonuclear cells and monocytes [1, 8, 9].

Various studies have shown that pretransplant immune status of the recipient can influence transplant outcome, acute rejection episodes, delayed graft function, and kidney survival. It was noted that pretransplant humoral autoimmunity (cross matches, panel reactive antibodies) affected graft outcome [10]. Previously, we as well as other authors have reported that cellular alloimmunity measured by the ELISPOT technique influenced the acute rejection rate and overall graft survival [11, 12].

In our population the acute rejection rate was less than 20%. Some studies observed that pretransplant blood cytokine pattern reflected immune infiltrates observed in kidney tissue during rejection. Similarly to some previous reports [7], we observed that none of the pretransplant cytokine patterns was associated with AR.

Th1 cytokines (IL-2, IFN-gamma) trigger an alloimmune response with activation of T cytotoxic lymphocytes, monocytes, and NK cells. Some author observed association between pretransplant Th1-derived cytokines and acute rejection episodes. Sadeghi et al. observed that high pretransplant IFN-gamma plasma levels were associated with acute rejection [13]. Pretransplant levels of anti-inflammatory cytokine TGF-beta tended to be lower among patients with acute rejection episodes, in contrast to upregulated Th1-derived cytokines [6]. Pretransplant serum levels of another proinflammatory cytokine, IL-6, were associated with an increased risk of acute rejection episodes and graft loss [14], and sIL-6R was higher and TGF-beta was lower in patients exhibiting ATN [15]. Unexpectedly, it was noted that pretransplant elevations of not only proinflammatory IL-12, but also anti-inflammatory cytokine IL-10, were both connected to acute rejection [16].

In contrast there are few reports of no relationship of pretransplant cytokine levels with acute rejection incidence. Chin et al. observed that pretransplant IFN-gamma levels did not predict acute rejection in a small retrospective study [17]. Also Ghafari et al. found no significant difference in serum concentration of IL-2, IL-10, IL-4, and IFN-gamma on the day before transplantation between the group with acute rejection within the first months and rejection-free recipients [7]. Sadeghi et al. did not observe any influence of pretransplant levels of examined cytokines IL-1, IL-2, IL-3, IL-4, IL-6, IL-10, TNF-alpha, TGF-beta, and IFN-gamma on acute rejection or ATN rate [15]. Among other conflicting reports we observed no correlation between immune factors' gene expression and acute rejection. Nevertheless in our study pretransplant gene expression of Th1-derived cytokines (*IL-2* and* IFNG*) significantly correlated with better posttransplant allograft function from the beginning up to 5 years. According to our knowledge, no previous papers have reported an association between pretransplant cytokine expression and kidney allograft function measured by GFR.

Th1-derived cytokines trigger an alloimmune response but also are easily suppressed by standard immunosuppressive drugs (calcineurin inhibitors act by inhibiting T cells through IL-2 deprivation). Furthermore, it is known that IFN-gamma inhibits synthesis of the potent rejection-maintaining cytokine IL-17 and further inhibits Th2 differentiation. This is a pathway of switching the alloresponse to a cellular rather than a humoral reaction. The humoral mechanism of rejection was recently described as more harmful and more difficult to manage than the cellular type. Graft failure has been described rarely after T-cell-mediated rejection and commonly after antibody-mediated rejection [18]. Moreover, some authors have observed elevated levels of IFN-gamma in stable patients after renal transplantation compared to an acute rejection group [13, 19]. That mechanism can partially explain our observation that higher pretransplant IFN-gamma expression was linked to better short- and long-term allograft function.

Apoptosis is an important mechanism of limiting immune response. We observed for the first time in the literature a positive correlation between pretransplant expression of three proapoptotic genes: for caspase-3, Fas, and IL-18 (*CASP3*,* IL-18*, and* FAS*), with allograft function expressed as eGFR. That association was seen not only in the early posttransplant period, but also in the long-term observation up to 5 years. Increased apoptosis of leukocytes was reported to be associated with increased proinflammatory activity in patients with chronic kidney disease. Fas, which is an apoptosis triggering cytokine, increased significantly with the stage of chronic kidney disease [20], especially in patients treated with peritoneal dialysis [21]. We did not find any published reports concerning expression of apoptosis-related genes in relation to further allograft function. In our study pretransplant expression of* TP53*, which belongs to the major apoptosis-inducing gene family, correlated negatively with WBC count. The majority of WBC are neutrophils. The chronic nonspecific immune reaction seen in uremia is mediated mainly by neutrophils [22]. The discovered positive correlation between proapoptotic gene expression and graft function may be partially explained by increased apoptosis of neutrophils and lymphocytes, which are known to be involved in the graft injury.

From the standard markers of inflammation, pretransplant CRP was reported to be a risk factor for acute rejection episodes [5] or not to influence graft function and survival among children [23] or adults [24]. In the present study we also found no influence of pretransplant CRP levels on the rate of delayed graft function or acute rejection and allograft function. After transplantation the restoration of renal function reduces the intensity of chronic inflammation in allograft recipients [25].

Severe pretransplant hypoalbuminemia was reported to be an independent risk factor for delayed graft function or graft loss [4, 26]. In contrast, we did not find higher risk of posttransplant DGF, AR, or worse graft function in hypoalbuminemic recipients, possibly because the majority of our recipients presented pretransplant albumin levels within the normal range. We noted that the albumin level correlated significantly with IL-10 gene expression in our patients. This is an expected finding because hypoalbuminemia was described as a potent factor augmenting chronic inflammation [27–29].

In our study recipients' pretransplant BMI negatively correlated with the short-term graft function from 3 to 12 months. Contrary results were published on the association of a higher 1-year GFR with pretransplant obesity and lower early GFR in patients with BMI < 18.5 kg/m^2^ [30]. More conflicting results on severe obesity and kidney allograft function were reported [31, 32]. Weissenbacher et al. found that recipient BMI correlated with the incidence of DGF and 1- and 5-year allograft function [33]. We did not find recipients' BMI to be a risk factor for acute rejection or DGF, probably because of low prevalence of obese and undernourished patients in our population (mean BMI was around 24 kg/m^2^).

Among donor-related issues, age is one of the most important factors negatively influencing outcomes of kidney allografts [34]. Like other authors, we found a strong correlation between donor age and kidney function from the beginning up to 2 years.

Apart from nonspecific time-related injury, kidneys from older donors were proved to be associated with a more intense host immune response early after transplant (up to 1 month) reflected by significantly higher numbers of peripheral T and B cells, increased T-cell alloreactivity, and modified cytokine patterns in rats [35]. Allografts from older donors were reported to be more immunogenic than those from younger donors [36, 37]. In our study, donor age was an independent significant risk factor not only for delayed graft function but also for acute rejection, which is in agreement with previous reports. This deleterious influence was still noticeable despite the fact that the majority of our recipients (86%) received grafts from donors younger than 60 years.

All the previously published results on the predictive value of pretransplant cytokine measurements were based on determination of cytokine blood concentration by ELISA technique. Our report for the first time assesses the cytokine gene expression in relation to posttransplant outcome. It was previously reported in a murine model that the IFN-gamma gene expression detected by real-time kinetic RT-PCR correlated very well with IFN-gamma protein secretion measured by ELISA [38]. Also Mohammadnia et al. examining serum levels of cytokines in kidney allograft recipients together with blood mRNA expression did not find any significant differences in those two ways of assessment of cytokine production [39]. Therefore our results reflect actual cytokine and other immune factors synthesis.

## 6. Conclusion

The study was performed to investigate whether pretransplant peripheral blood immune gene expression affects clinical outcome of renal allograft. The lowered pretransplant Th1-derived cytokine and apoptosis-related gene expressions were a hallmark of subsequent worse kidney function but not of acute rejection rate. The pretransplant* IFNG* and* CASP3* and* FAS* and* IL-18* genes expression in the recipients' peripheral blood is the possible candidate for novel biomarker of short- and long-term allograft function.

## Figures and Tables

**Table 1:** Donor and recipient characteristics (mean ± SD or number of cases).

*Recipients*	
Recipient age (years)	47 ± 14
Recipient gender (female/male)	34/53
Dialysis (HD/PD)	66/21
Time of dialysis (months)	33 ± 42
BMI (kg/m^2^)	24.6 ± 3.6
Last PRA >20%	3
*Primary kidney disease*	
Diabetic nephropathy	9
Chronic glomerulonephritis	34
Hypertensive nephropathy	16
Polycystic renal disease	10
Chronic interstitial nephritis	14
Other/unknown	4
*Donors*	
Donor age (years)	45 ± 13
Donor gender (female/male)	34/53
CIT (hours)	25.1 ± 6.9
*Number of HLA mismatches *	
0	2
1	1
2	13
3	28
4	33
5	9
6	1

**Table 2:** Pretransplant recipient blood parameters.

	Mean ± SD
Hgb (g/dL)	11.1 ± 1.7
WBC count (×10^3^/mcL)	7.4 ± 2.3
PLT count (×10^3^/mcL)	198 ± 64
CRP (mg/L)	6.9 ± 7.0
Cholesterol (mg/dL)	181 ± 57
Albumin (g/dL)	4.2 ± 0.8
Creatinine (mg/dL)	7.0 ± 2.0
Uric acid (mg/dL)	5.4 ± 2.8

**Table 3:** Expression level of the studied genes.

Gene	Min.	25%	Median	75%	Max.	Mean
*CASP3*	−0.78	0.42	1.17	1.85	3.52	1.17
*FAS*	−0.68	0.39	0.87	1.33	2.31	0.83
*FOXP3*	−0.6	0.37	0.91	1.37	2.31	0.88
*IFNG*	−1.71	0.7	1.72	2.95	5.38	1.82
*IL-10*	−0.66	0.34	0.7	1.26	2.74	0.82
*IL-18*	−0.75	0.34	0.68	1.12	2.68	0.71
*IL-2*	−1.33	0.54	1.21	2.78	7.37	1.88
*IL-6*	−3.02	1.03	1.79	2.74	4.4	1.73
*IL-8*	−1.18	0.63	1.41	2.21	4.5	1.44
*LCN2*	−0.92	0.73	1.61	2.65	4.85	1.72
*TGFB1*	−0.43	0.27	0.41	0.67	1.25	0.43
*TNF*	−0.56	0.36	0.67	0.97	1.56	0.63
*TP53*	−1	0.25	0.57	0.88	1.42	0.55

**Table 4:** Significant correlations between pretransplant clinical parameters and gene expression.

Clinical parameter	Gene expression	Correlation coefficient	*P*
WBC	*FOXP3 *	−0.30	0.020
	*IL-2* ^*∗*^	−0.32	0.014
	*LCN2 *	0.28	0.026
	*TNF *	−0.34	0.008
ALB	*IL-10 *	0.27	0.025
HGB	*IL-8 *	0.29	0.021
BMI	*LCN2 *	0.45	<0.001
Recipient age	*TP53 *	−0.26	0.014

^*∗*^Spearman correlation *r*_*s*_ and probability.

**Table 5:** Correlation of gene expression and pretransplant clinical parameters with GFR—simple and multiple linear regression analysis.

	Simple regression		Multiple regression^*∗*^	
	Corr. coeff.	*P*	Corr. coeff	*P*
*14-day GFR*				
Recipient age	−0.33	0.003	**−0.27**	**0.012**
Donor age	−0.33	0.004	**−0.29**	**0.008**
* TGFB1*	−0.24	0.039	**−0.25**	**0.016**

*1-month GFR*	** **	** **	** **	** **
Recipient age	−0.37	0.001	**−0.29**	**0.005**
Donor age	−0.41	0.000	**−0.34**	**0.001**

*3-month GFR*	** **	** **	** **	** **
Recipient age	−0.31	0.010	−0.18	0.176
Donor age	−0.44	0.000	**−0.29**	**0.028**
BMI	−0.35	0.009	−0.26	0.065
* LCN2*	−0.27	0.024	−0.15	0.256

*6-month GFR*	** **	** **	** **	** **
Recipient age	−0.39	0.001	**−0.27**	**0.018**
Donor age	−0.60	0.000	**−0.40**	**0.000**
BMI	−0.36	0.010	**−0.38**	**0.001**
* CASP3*	0.27	0.027	0.20	0.072
* FAS*	0.28	0.021	0.09	0.405
* IFNG*	0.27	0.029	**0.32**	**0.003**

*12-month GFR*	** **	** **	** **	** **
Recipient age	−0.33	0.008	**−0.22**	**0.047**
Donor age	−0.48	0.000	**−0.42**	**0.000**
* CASP3*	0.30	0.016	**0.23**	**0.038**
* IFNG*	0.30	0.018	**0.23**	**0.034**

*24-month GFR*	** **	** **	** **	** **
Recipient age	−0.35	0.012	−0.20	0.082
Donor age	−0.55	0.000	**−0.50**	**0.000**
* CASP3*	0.41	0.003	**0.34**	**0.003**
* FAS*	0.30	0.033	**0.23**	**0.046**
* IFNG*	0.29	0.040	0.20	0.086
* IL-18*	0.28	0.044	**0.23**	**0.047**

^*∗*^Correlation coefficient for a clinical parameter was corrected for other clinical parameters and all listed genes influencing GFR at a given time point; each gene expression was corrected for all clinical parameters influencing GFR at a given time point.

**Table 6:** ROC analysis results for graft function (GFR > 50 mL/min) 24 months after KTx.

Gene	Cut-off	Sensitivity	Specificity	AUC	*P*
*CASP3*	1.613	0.71	0.88	0.79	0.0002
*FAS*	1.150	0.64	0.80	0.75	0.0029
*IL-18*	0.781	0.71	0.80	0.77	0.0011

## Abbreviations

AR: Acute rejection
BMI: Body mass index
CIT: Cold ischemia time
DGF: Delayed graft function
eGFR: Estimated glomerular filtration rate
ELISPOT: Enzyme-Linked ImmunoSpot Assay
*FAS*: First apoptotic signal
*FOXP*3: Forkhead box P3
GAPDH: Glyceraldehyde 3-phosphate dehydrogenase
HD: Hemodialysis
HLA: Human leucocyte antigens
IFN-gamma: Interferon gamma
IL-10: Interleukin 10
IL-18: Interleukin 18
IL-2: Interleukin 2
IL-6: Interleukin 6
IL-8: Interleukin 8
NGAL: Neutrophil gelatinase-associated lipocalin
p53: Tumor protein p53
PCR: Polymerase chain reaction
PD: Peritoneal dialysis
PRA: Panel reactive antibodies
TGF-beta: Transforming growth factor beta
TNF-alpha: Tumor necrosis factor alpha
WBC: White blood cell.
